# Activated Pancreatic Stellate Cells Enhance the Warburg Effect to Cause the Malignant Development in Chronic Pancreatitis

**DOI:** 10.3389/fonc.2021.714598

**Published:** 2021-09-03

**Authors:** Ye Tao, Feng Shao, Ming Cai, Zhen Liu, Yao Peng, Qiang Huang, Futao Meng

**Affiliations:** ^1^Department of General Surgery, Anhui Provincial Hospital Affiliated of Anhui Medical University, Hefei, China; ^2^Department of General Surgery, The First Affiliated Hospital of University of Science & Technology of China, Anhui Provincial Hospital, Hefei, China; ^3^Department of Oncology, The Second Affiliated Hospital of Anhui Medical University, Hefei, China; ^4^Department of Surgical Oncology, The First Affiliated Hospital of Bengbu Medical College, Bengbu, China

**Keywords:** activated pancreatic stellate cells, co-culture, the Warburg effect, malignant development in chronic pancreatitis, pancreatic ductal adenocarcinoma

## Abstract

Chronic pancreatitis (CP) is a precancerous condition associated with pancreatic ductal adenocarcinoma (PDAC), but its evolutionary mechanism is unclear. Pancreatic stellate cells (PSCs) are closely related to the occurrence and development of CP and PDAC, but it is not clear whether PSCs play a key role in this “inflammation-cancer transition”. Our research found that co-culture with activated PSCs promoted the proliferation, migration and invasion of normal pancreatic duct epithelial cells and pancreatic cancer cells. At the same time, activated PSCs had a significant effect on the expression of the glycolysis markers (pyruvate kinase M2, lactate dehydrogenase A, glucose transporter 1, hexokinase-II and monocarboxylate transporter 4; PKM2, LDHA, GLUT1, HK2 and MCT4) in normal pancreatic duct epithelial cells and pancreatic cancer cells and increased lactic acid production and glucose consumption in these two cells. *In vivo* experiments showed that the expression of the glycolysis markers in pancreatic duct epithelial cells and the marker protein (α-SMA) of activated PSCs in the pancreatic duct peripancreatic interstitium were higher in pancreatic cancer tissues and chronic pancreatitis tissues than in normal pancreatic tissues in both animals and humans. In addition, analysis of human tissue specimens showed that there is a correlation between the expression of glycolysis markers and α-SMA. These findings indicate that activated PSCs play an important role in the development and progression of chronic pancreatitis into pancreatic cancer by regulating and promoting aerobic glycolysis. Our research provides a new theoretical basis for further understanding the mechanism of CP malignancy and the selection of targets for reversing CP malignancy.

## Introduction

Pancreatic cancer is a highly malignant tumor with a poor prognosis. Its mortality ranked seventh among all malignant tumors in 2020, and its incidence is increasing annually (1).Early pancreatic cancer lacks specific clinical symptoms. Nearly 85% of patients have lost the chance for radical surgical resection by the time they consult a doctor, and their 5-year survival rate is less than 6%. Even after radical surgery, the 5-year survival rate of patients is less than 25% (2). Understanding the mechanism of pancreatic cancer will help to guide research on the prevention, early diagnosis and treatment of pancreatic cancer. The literature points out that pancreatic cancer is related to smoking, drinking, obesity, age, chronic pancreatitis, etc. (3). Among these factors, chronic pancreatitis is considered a precancerous condition associated with pancreatic cancer. Chronic pancreatitis is an inflammatory process in which the endocrine and exocrine glands of the pancreas are progressively and irreversibly damaged and the pancreatic parenchyma is replaced by fibrous tissue, which ultimately leads to various morphological and functional changes in the pancreas and triggers a series of clinical signs of inflammation (4). Clinical studies have shown that patients with chronic pancreatitis have a much higher risk of pancreatic cancer than normal people (5, 6). Therefore, exploring the mechanism of pancreatic cancer transformation from chronic pancreatitis and reversing the progression of chronic pancreatitis are important issues that urgently need to be solved.

Pancreatic stellate cells (PSCs) are pancreatic-specific mesenchymal cells that accumulate around blood vessels and ducts in pancreatic tissue and surround the base of acini. PSCs only account for approximately 4% of pancreatic cells in the normal quiescent state; their cytoplasm contains abundant lipid droplets containing vitamin A, and they are characterized by the expression of GFAP and desmin (7–9). PSCs are considered to be the targets of various cytokines in chronic pancreatitis and the core of the progression of chronic pancreatitis. PSCs are activated during pancreatic tissue injury and stress and acquire a myofibroblast phenotype. Lipid droplets rich in vitamin A disappear in their cytoplasm. With the expression of α-smooth muscle actin (α-SMA) as a marker, a large amount of extracellular matrix (ECM) is synthesized, and a variety of cytokines are secreted, to promote irreversible pancreatic fibrosis in chronic pancreatitis (10, 11). During the activation of PSCs, α-SMA and lipid droplets are recognized as markers of activated and quiescent PSCs, respectively (9). Not only are PSCs the initiating factors of chronic pancreatitis, a large number of reports also indicate that they play an important role in the occurrence, development and metastasis of pancreatic cancer (12–14). However, whether PSCs play an important role in the malignant transformation of chronic pancreatitis to PDAC has not been reported. To maintain their abnormal proliferation ability and enhanced invasion characteristics, tumor cells reprogram nutrient transporters and glucose metabolism enzymes to maintain a high level of metabolism. This metabolic characteristic of cancer cells is manifested by a greatly increased rate of glucose uptake and very active glycolysis even in the case of sufficient oxygen supply. This phenomenon is called aerobic glycolysis (the Warburg effect) (15). Currently, some studies have confirmed that PSCs promote glycolytic metabolism in pancreatic cancer (16, 17), but whether PSCs can promote the malignant transformation of CP by affecting the Warburg effect has not been reported.

In this study, we first isolated and cultured PSCs from human pancreatic cancer and normal pancreatic tissues and then performed phenotypic identification. We successfully obtained activated PSCs (derived from PDAC tissue, CaPSCs) and quiescent PSCs (derived from normal pancreas tissue, NaPSCs). Subsequently, we co-cultured PSCs from different sources with pancreatic cancer cells/normal pancreatic duct epithelial cells and found that co-culture promoted the proliferation, migration and invasion of these two cell types. Finally, we tested the expression of glycolysis markers and glycolysis metabolism levels related to the Warburg effect in pancreatic cancer cells/normal pancreatic duct epithelial cells and further confirmed these experimental results *in vivo*. Our findings suggest that activated PSCs can enhance the Warburg effect to promote the malignant transformation of CP. The specific mechanism needs further research.

## Materials and Methods

### Patients and Tissue Samples

Primary pancreatic ductal adenocarcinoma tissue, chronic pancreatitis tissue and normal pancreatic tissue were obtained from 40 patients with pancreatic head cancer, 19 patients with chronic pancreatitis and 20 patients with duodenal benign tumors between January 2012 and January 2018 at the First Affiliated Hospital of University of Science and Technology of China. All tissue specimens were fixed in formalin for 24 h and then embedded in paraffin. The study was approved by the Ethics Committee of the Anhui Provincial Hospital of the First Affiliated Hospital of the University of Science and Technology of China, and informed consent was obtained from each participant.

### Cell Lines and Cell Culture

PANC-1 cells were obtained from Shanghai Cell Bank. hTERT-HPNE cells (a human normal pancreatic ductal epithelial cell line) were obtained from the American Type Culture Collection. PANC-1 cells were cultured in DMEM (HyClone Laboratories Inc, USA) containing 10% fetal bovine serum (Biological Industries, ISR). hTERT-HPNE cell lines were cultured in the recommended complete growth medium, which included 5% fetal bovine serum, 75% DMEM without glucose, 25% Medium M3 Base, 10 ng/mL human recombinant EGF, 5.5 mmol/L D‐glucose (1 g/L) and 750 ng/mL puromycin. We collected surgical resection of PDAC tissues from 5 patients with pancreatic head cancer and normal pancreatic tissues from 5 patients with benign space-occupying duodenum between January 2019 and December 2019 in the Department of Biliary and Pancreatic Surgery of the First Affiliated Hospital of University of Science and Technology of China and then used a previous method to isolate and culture PSCs (18). Then, CaPSCs derived from pancreatic cancer and NaPSCs derived from normal pancreas were obtained. All cell lines were cultured in a 37°C, 5% CO2 incubator. The PSCs used in cell culture experiments were passaged 2-8 times. Transwell co-culture system (6-well plate, 0.4μm, 3412#, Corning, USA) was used. The upper chamber was seeded with 3×10^5^ PANC-1 or hTERT-HPNE, and the lower plate was seeded with 3×10^5^ human pancreatic stellate cells from different sources in a 5% incubator at 37°C for 24 h.

### Immunofluorescence

The specific operation process was the same as before (18). Isolated PSCs from different sources which were passaged 2-3 times were inoculated into a six-well culture plate equipped with a cover slip, cultured under standard conditions for 48 hours, and then incubated with primary antibody (rabbit monoclonal anti-α-SMA, 1:100, Abcam, USA). The cells were detected and imaged with a fluorescence microscope, and the purity of PSCs was analysed.

### Oil Red O Staining

Add 2 mL of PBS (preheated at 37°C) to the 6-well plate inoculated with PSCs from different sources which were passaged 2-3 times and shake gently. After the adherent cells were washed twice with PBS, the remaining buffer was removed. The PSCs were fixed in 1 mL formaldehyde solution (100 g/L) at room temperature (RT) for 15 min. After removing the formaldehyde solution, the fixed PSCs were washed twice with PBS. Subsequently, 3 mL of newly prepared Oil Red O working solution was slowly, and then the PSCs were observed under an inverted microscope. Once the fat droplets appear in the form of bright red beads of different sizes, absorb the oil red O working solution to stop staining. Rinse with PBS for 2 to 3 times, and counterstain with hematoxylin for 30 seconds. Then, the PSCs were washed again with PBS, and subjected to differentiation/decolorization. The PSCs were imaged under a microscope.

### Cell Proliferation Assay

PANC-1 and HPNE cells co-cultured with PSCs from different sources were routinely digested and seeded in a 96-well plate at 3000 cells per well. The Cell Counting Kit-8 (CCK-8, Beyotime, Shanghai, China) method was used to detect the proliferation of pancreatic cancer cells, and cells that were not co-cultured were used as controls. Briefly, premixed medium (10 μL of CCK-8 solution, 100 μL of medium) was added to each well, and after incubation in the dark at 37°C for 3 h, a microplate reader was used to measure the light absorption value at 450 nm to evaluate cell viability.

### Migration and Invasion Assays

The effect of activated PSCs on the migration and invasion of pancreatic ductal cells was evaluated using Transwell chambers. CaPSCs and NaPSCs were inoculated in a 24-well plate (24-well plate, 8μm, 3422#, Corning, USA) at a density of 5×104 cells/well. After 24 h, the medium was replaced with 750 μL of 10% FBS DMEM. The chambers were placed in a 24-well culture dish, and HPNE (2×10^4^) and PANC-1 (2×10^4^) cells resuspended in 200 μL of serum-free medium were then seeded into the upper compartments containing either uncoated or Matrigel-coated membranes. After incubation for 24 h, the compartments were removed for cleaning, and cells on the lower surface were fixed with methanol and stained with crystal violet. The migrated cells on the lower surface were counted to assess migration and invasion.

### RNA Isolation and Quantitative Real‐Time PCR

Cellular RNA was extracted by an RNA extraction kit (Takara, Japan), and cDNA was synthesized by a reverse transcription kit (Takara, Japan). qPCR was performed on a real-time PCR system (Roche, Switzerland) using a SYBR Green PCR kit(Takara, Japan). All primers (**Table 1**) were synthesized by Sangon Biotech (Shanghai, China). The RNA levels were normalized to human ACTB(β-actin) levels.

**Table 1:** Primer sequence.

Gene name	Primer sequences, 5′-3′
PKM2	F: TGACGAGAACATCCTGTGGC
	R: AGCACAGATGACAGGCTTCC
LDHA	F: GCCTGTATGGAGTGGAATGAA
	R: CCAGGATGTGTAGCCTTTGAG
GLUT1	F: AACTCTTCAGCCAGGGTCCA
	R: CACAGTGAAGATGATGAAGAC
HK2	F: GATTGTCCGTAACATTCTCATCGA
	R: TGTCTTGAGCCGCTCTGAGAT
MCT4	F: CCAGGCCCACGGCAGGTTTC
	R: GCCACCGTAGTCACTGGCCG
ACTB	F: CTGGAACGGTGAAGGTGACA
	R: AAGGGACTTCCTGTAACAACGCA

### Western Blotting

Briefly, RIPA lysis buffer (Beyotime, Shanghai, China) was applied to fully lyse the cells and extract total protein. After the protein concentration was determined, loading buffer (1:4, Beyotime) was added and then denatured in boiling water. SDS-PAGE was used after protein loading. After electrophoresis, proteins on the gel were transferred to PVDF membranes by the wet transfer method. After full sealing with the blocking solution, the membrane was transferred to a primary antibody (PKM2, LDHA rabbit polyclonal antibody, Abcam, USA; GLUT1, HK2, MCT4 rabbit polyclonal antibody, Proteintech, China) diluted 1:1000 and incubated at 4°C overnight. After washing the membrane, it was transferred to the secondary antibody diluted at an appropriate proportion and incubated at room temperature for 2 h. After washing the membrane again, immunoreactions were visualized by chemiluminescence.

### Lactate Production and Glucose Utilization Assays

Human pancreatic stellate cells (3×10^5^ cells/well) from different sources were seeded into 6-well plates for later use. Transwell co-culture system (6-well plate, 0.4μm, 3412#, Corning, USA) was used. The upper chamber was seeded with 3×10^5^ PANC-1 or HPNE, while the lower chamber was not seeded with cells. After 12h culture with 10%FBS medium, the cells were replaced with serum-free medium for starvation overnight. Subsequently, the upper chambers were moved to six-well plates pre-inoculated with human pancreatic stellate cells from different sources, and co-cultured for 24h. According to the instructions, a glucose detection kit and lactic acid detection kit (Jiancheng Bioengineer Institute, Nanjing, China) were used to detect the glucose and lactic acid contents in the culture medium of HPNE and PANC-1 cells co-cultured from different PSC sources, and normalization treatment was conducted according to the number of cells. Three replicates were established for each group, and the experiment was repeated three times. Glucose intake = (glucose content in the original medium - glucose content in the medium after 24 h of co-culture)/number of cells; lactic acid secretion = (lactic acid content in the medium after 24 h of co-culture - lactic acid content in the original medium)/number of cells.

### Animals and Animal Models

Adult male SD rats weighing 180–200 g were used. They were maintained in accordance with the guidelines of the Committee on Animal Care of the First Affiliated Hospital of University of Science and Technology of China. Twenty rats in the CP model group were injected with Dibutyltin dichloride (DBTC, Sigma, Germany) solution (8 mg/mL/kg) through the tail vein, and six rats in the control group were injected with an equal volume of normal saline. After 6 weeks of modelling, the rats were anaesthetized and killed by cervical dislocation. Twenty rats in the PDAC model group were fasted for 24 h before surgery (water was provided). After intraperitoneal injection of 3% pentobarbital sodium 1.5 ml/kg, the pancreas was exposed through a median incision in the upper abdomen, and then the pancreatic capsule was cut open at the flat and firm part of the pancreas. The model group was implanted with 6 mg 7,12-Dimethylbenz[a]anthracene (DMBA, Sigma, Germany), and six rats in the control group underwent no other operations. Then, the abdomens were closed after the capsules were sutured. After 16 weeks of modelling, the rats were anaesthetized and then killed by cervical dislocation. All tissue specimens were fixed with formalin for 24 h and then embedded in paraffin. The HE staining sections were imaged using an inverted microscope (BX51, OLYMPUS, Japan). The animal models were confirmed by pathological diagnosis.

### Immunohistochemistry

The paraffin-embedded sections were dewaxed with xylene, hydrated with gradient ethanol and boiled in citrate buffer for 20 min. Endogenous peroxidase activity was blocked by 3% H2O2. Rabbit polyclonal antibodies (PKM2, LDHA rabbit polyclonal antibody, Abcam, USA; GLUT1, HK2, MCT4 rabbit polyclonal antibody, Proteintech, China) were added at a concentration of 1:100 and incubated overnight at 4°C. After incubation with the secondary antibody for 1 hour, DAB was used for color development. Finally, the sections were re-stained with hematoxylin and fixed by dehydration. For the negative control group, the main antibodies were replaced with PBS under the same conditions. The HE staining/immunohistochemical sections were imaged using an inverted microscope (BX51, OLYMPUS, Japan). Semi-quantitative method was used to score the results: staining area: 0, no staining; 1 point, 10%; 2 points, 10%-30%; 3, 30% staining; Dyeing intensity: 0, none; 1 points, light yellow; 2 points, yellow; 3 points, brown and yellow. The total score was the product of the staining area and staining intensity score. Low expression was defined as a total score ≤3, and high expression was defined as a total score > 3. All immunohistochemical slides were scored independently by two experienced pathologists, and differences were adjusted for by a joint assessment.

### Statistical Analysis

Statistical data are presented as the means ± SDs (n = 3). Student’s t test, chi-square test and Spearman correlation analysis were used for statistical analysis. P values of <0.05 were accepted as statistically significant.

## Results

### Immunofluorescence and Oil Red O Staining Show That CaPSCs Had the Characteristics of Activated PSCs and NaPSCs Had the Characteristics of Quiescent PSCs

**Figure 1:**
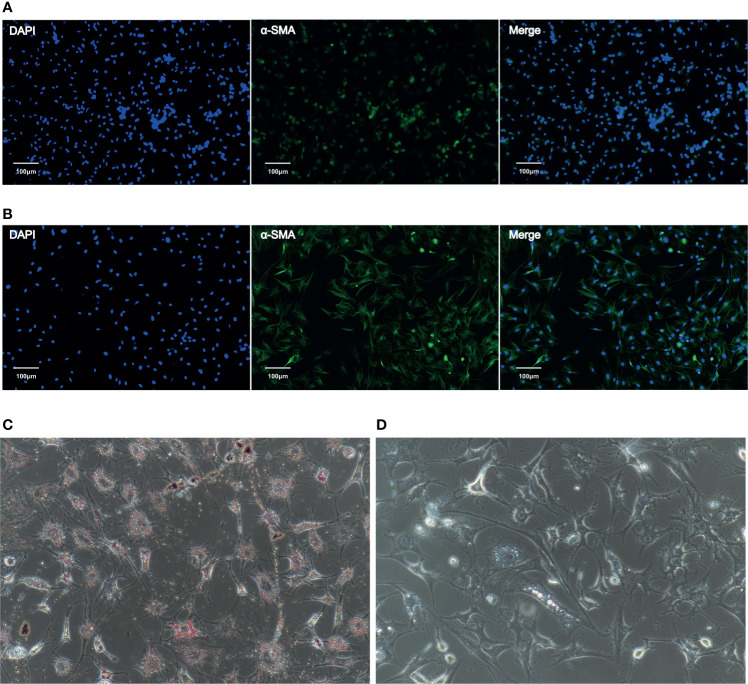
The expression of α-SMA in NaPSCs and CaPSCs was detected by immunofluorescence, and CaPSCs showed a highly activated state. **(A)** NaPSC immunofluorescence—DAPI, α-SMA, MERGE; **(B)** CaPSC immunofluorescence—DAPI, α-SMA, MERGE; **(C)** NaPSC Oil red O staining **(D)** CaPSC Oil red O staining.

Phenotypic identification of CaPSCs was performed by immunofluorescence labelling of α-SMA, a marker protein of activated pancreatic stellate cells. Although quiescent PSCs will gradually activate and express α-SMA *in vitro*, immunofluorescence can distinguish them from activated PSCs. The expression of α-SMA in CaPSCs (**Figure 1A**) and their phenotypic characteristics were significantly different from NaPSCs (**Figure 1B**), indicating that CaPSCs had the characteristics of activated PSCs. Oil red O staining was used to detect the presence of lipid droplets in quiescent PSCs. Red lipid droplets can be observed in NaPSCs (**Figure 1C**), but no lipid droplets in CaPSCs (**Figure 1D**).

### Co-Culture With CaPSCs Promotes the Proliferation, Migration, and Invasion of Benign and Malignant Pancreatic Duct Epithelial Cells

**Figure 2:**
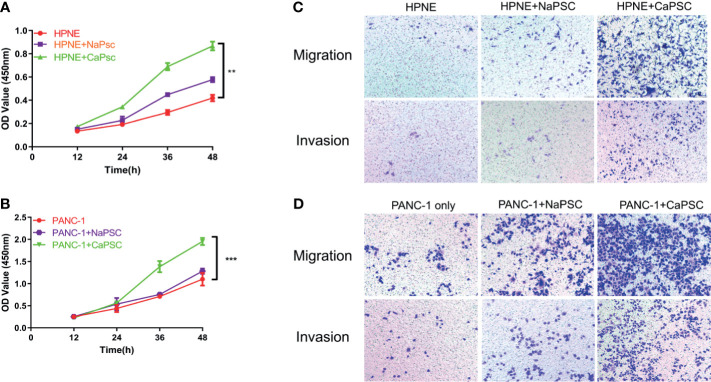
Co-culture with CaPSCs enhanced the proliferation, invasion and migration of HPNE and PANC-1 cells. **(A)** CCK-8 proliferation assay after co-culture of HPNE cells with NaPSCs or CaPSCs. **(B)** CCK-8 proliferation assay after co-culture of PANC-1 cells with NaPSCs or CaPSCs. **(C)** Transwell migration and invasion assays after co-culture of HPNE cells with NaPSCs or CaPSCs. **(D)** Transwell migration and invasion assays after co-culture of PANC-1 cells with NaPSCs or CaPSCs. **P < 0.01, ***P < 0.001.

The CCK-8 proliferation assay showed that the proliferation ability of HPNE (**Figure 2A**) and PANC-1 (**Figure 2B**) cells in the CaPSC co-culture group was significantly improved compared with that in the blank groups (P<0.01; P<0.001). Transwell experiments showed that the migration and invasion abilities of HPNE and PANC-1 cells in the CaPSC co-culture group were significantly improved compared with those in the NaPSC and blank groups. (**Figures 2C, D**)

### Co-Culture With CaPSCs Promotes the Warburg Effect in Pancreatic Duct Epithelial Cells (Increased Expression of PKM2, LDHA, GLUT1, HK2 and MCT4; Increased Lactic Acid Production and Glucose Consumption)

**Figure 3:**
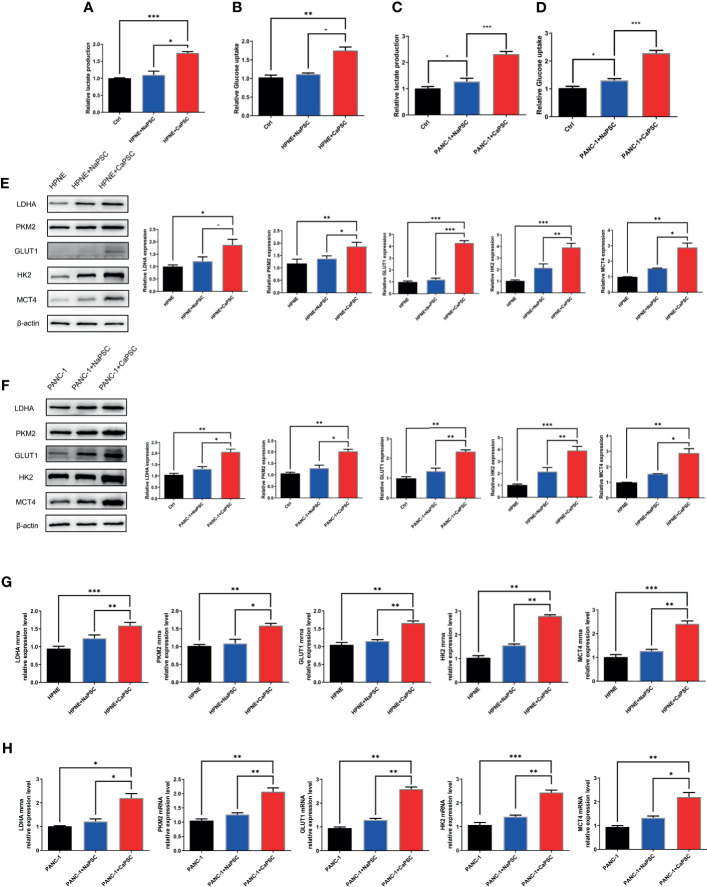
Co-culture with CaPSCs promoted lactic acid production, glucose consumption and the expression of the glycolytic proteins LDHA/PKM2 in HPNE and PANC-1 cells. **(A)** Changes in HPNE cell lactic acid production after co-culture with NaPSCs or CaPSCs; **(B)** Changes in HPNE cell glucose consumption after co-culture with NaPSCs or CaPSCs; **(C)** Changes in PANC-1 cell lactic acid production after co-culture with NaPSCs or CaPSCs; **(D)** Changes in PANC-1 cell glucose consumption after co-culture with NaPSCs or CaPSCs; **(E)**, E1-E5; G1-G5: Changes in the protein and mRNA expression of PKM2, LDHA, GLUT1, HK2 and MCT4 in HPNE cells after co-culture with NaPSCs or CaPSCs; **(F)**, F1-F5; H1-H5: Changes in the protein and mRNA expression of PKM2, LDHA, GLUT1, HK2 and MCT4 in PANC-1 cells after co-culture with NaPSCs or CaPSCs; *P < 0.05, **P < 0.01, ***P < 0.001.

Lactate production and glucose utilization assays showed that the lactic acid production (**Figure 3A**) and glucose consumption (**Figure 3B**) of HPNE cells in the CaPSC group were significantly higher than those in the blank group (P<0.001; P<0.01); the lactic acid production (**Figure 3C**) and glucose consumption (**Figure 3D**) of PANC-1 cells in the CaPSC group were significantly higher than those in the blank group(P<0.001; P<0.001). Western blotting showed that the protein expression levels of PKM2 (**Figure 3, E1**), LDHA (**Figure 3, E2**), GLUT1 (**Figure 3, E3**), HK2 (**Figure 3, E4**) and MCT4 (**Figure 3, E5**) in HPNE cells in the CaPSC co-culture group were significantly increased compared with those in the blank groups(P<0.05; P<0.01; P<0.001; P<0.001; P<0.01); the protein expression levels of PKM2 (**Figure 3, F1**), LDHA (**Figure 3, F2**), GLUT1 (**Figure 3, F3**), HK2 (**Figure 3, F4**) and MCT4 (**Figure 3, F5**) in PANC-1 cells in the CaPSC co-culture group were significantly increased compared with those in the blank groups (P<0.01; P<0.01; P<0.01; P<0.001; P<0.01). Quantitative real‐time PCR showed that the mRNA expression levels of PKM2 (**Figure 3, G1**), LDHA (**Figure 3, G2**), GLUT1 (**Figure 3, G3**), HK2 (**Figure 3, G4**) and MCT4 (**Figure 3, G5**) in HPNE cells in the CaPSC co-culture group were significantly increased compared with those in the blank groups (P<0.001; P<0.01; P<0.01; P<0.01; P<0.001); the mRNA expression levels of PKM2 (**Figure 3, H1**), LDHA (**Figure 3, H2**), GLUT1 (**Figure 3, H3**), HK2 (**Figure 3, H4**) and MCT4 (**Figure 3, H5**) in PANC-1 cells in the CaPSC co-culture group were significantly increased compared with those in the blank groups (P<0.05; P<0.01; P<0.01; P<0.001; P<0.01).

### Establishment of CP and PDAC Rats; Immunohistochemical Analysis Showing the Activation of PSCs and the Enhancement of Glycolysis in Diseased Tissues

**Figure 4:**
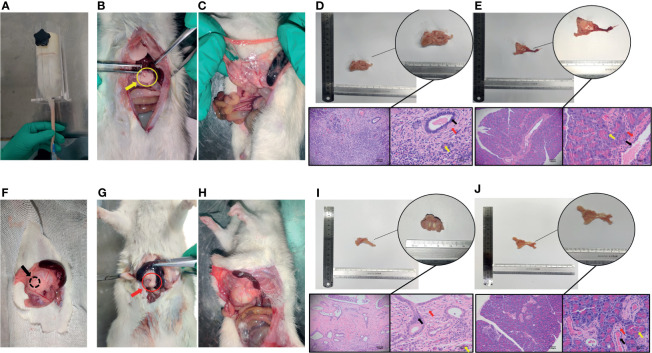
Establishment and identification of CP and PDAC model rats. **(A)** The rat CP models were established by injecting DBTC solution through the tail vein; **(B, C)** After 6 weeks of modelling, the pancreas of rats in the CP group and the pancreas of rats in the control group (injection of saline) were collected. Severe fibrosis was observed in the pancreas - yellow arrow of the CP group; **(F)** Rats with orthotopic pancreatic cancer induced by surgical implantation of DMBA; **(G, H)**: Sixteen weeks after modelling, the pancreas of rats *in situ* PDAC and the pancreas of rats in the sham operation control group. Severe fibrosis and local mass formation were observed at the pancreatic red arrow of the rats in the *in situ* PDAC group; **(D, E)** Gross pancreatic specimens and pathological HE staining of rats in the CP group and control group (100X and 400X; black arrow: normal epithelial duct cells; red arrow: stromal cells (fibroblasts, PSCs); yellow arrow: acinar cells); **(I, J)** Gross pancreatic specimens and pathological HE staining of rats in the *in situ* PDAC group and control group (100X and 400X; black arrow: cancer/normal epithelial duct cells; red arrow: stromal cells (fibroblasts, PSCs); yellow arrow: acinar cells).

**Figure 5:**
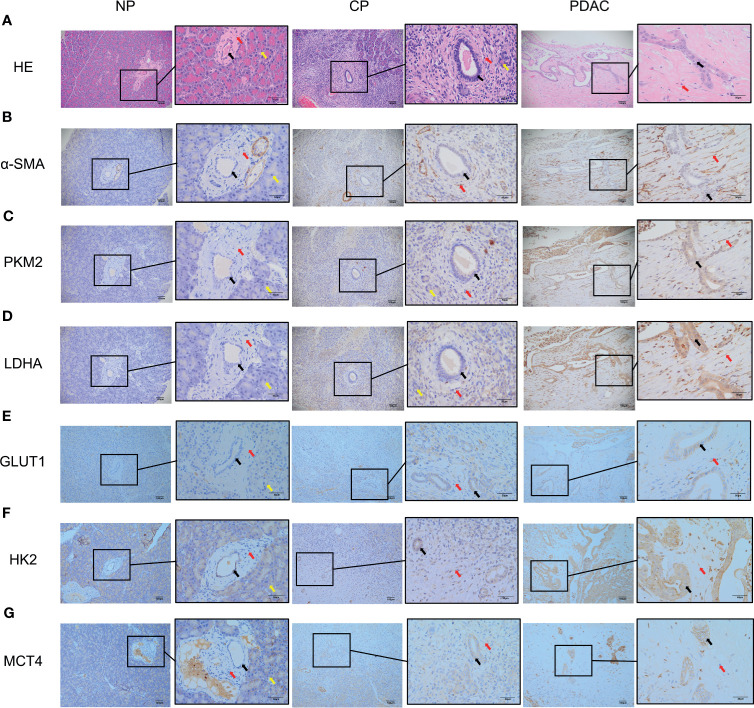
Immunohistochemistry showed that the α-SMA and glycolysis markers in the pancreatic tissues of rats in the normal group, CP group and PDAC group gradually increased (100X and 400X; black arrow: normal/cancer epithelial duct cells; red arrow: stromal cells (fibroblasts, PSCs); yellow arrow: acinar cells). **(A)** HE in normal/CP/PDAC rat pancreas; **(B)** α-SMA in normal/CP/PDAC rat pancreas; **(C)** PKM2 in normal/CP/PDAC rat pancreas; **(D)** LDHA in normal/CP/PDAC rat pancreas; **(E)** GLUT1 in normal/CP/PDAC rat pancreas; **(F)** HK2 in normal/CP/PDAC rat pancreas; **(G)** MCT4 in normal/CP/PDAC rat pancreas.

The CP rat model (**Figures 4B, D**) was established by DBTC caudal intravenous injection (**Figure 4A**) , and the incidence of CP in rats was 35% after 6 weeks, and the pancreas of rats in the control group (**Figures 4C, E**) injected with normal saline were normal; the PDAC rat model (**Figures 4G, I**) was established using DMBA surgical implantation into the pancreas (**Figure 4F**), and the incidence of PDAC in rats was 30% after 16 weeks, and the pancreas of rats in the surgical control group (**Figures 4H, J**) were normal. The statistical results of animal model establishment are briefly listed in **Table 2** and **Table 3**. Subsequently, paraffin sections of CP and PDAC pancreatic tissues were stained with α-SMA and glycolysis markers (PKM2, LDHA, GLUT1, HK2 and MCT4) immunohistochemistry. The results showed that these glycolysis markers were not expressed or weakly expressed in pancreatic duct epithelial cells and that α-SMA was weakly expressed in peri-pancreatic duct stroma in normal pancreatic tissue of rats. These glycolysis markers in pancreatic duct epithelial cells and α-SMA in peripancreatic duct stroma were higher in the CP group than in the normal pancreas group. This phenomenon has also been observed in rat pancreatic cancer tissues, The protein expression at these sites appeared to be higher than in the CP group (**Figures 5A–G**).

**Table 2:** Statistical results of CP animal model.

	DBTC	NS control
CP	7(35.0%)	0(0%)
Pancreatic edema/hemorrhage only	5(25.0%)	0(0%)
Death	2(10.0%)	0(0%)
Normal pancreas	6(30.0%)	6(100%)
Total	20(100%)	6(100%)

**Table 3:** Statistical results of PDAC animal model.

	DMBA	Surgical control
PDAC	6(30.0%)	0(0%)
PanIN	3(15.0%)	0(0%)
Pancreatic edema/hemorrhage only	5(25.0%)	1(16.7%)
Death	3(15.0%)	1(16.7%)
Normal pancreas	1(5.0%)	4(66.6%)
Total	20(100%)	6(100%)

### Immunohistochemical Analysis of Human CP and PDAC Tissues Shows That the Activation of PSCs Is Correlated With the High Expression of Glycolytic Proteins

**Figure 6:**
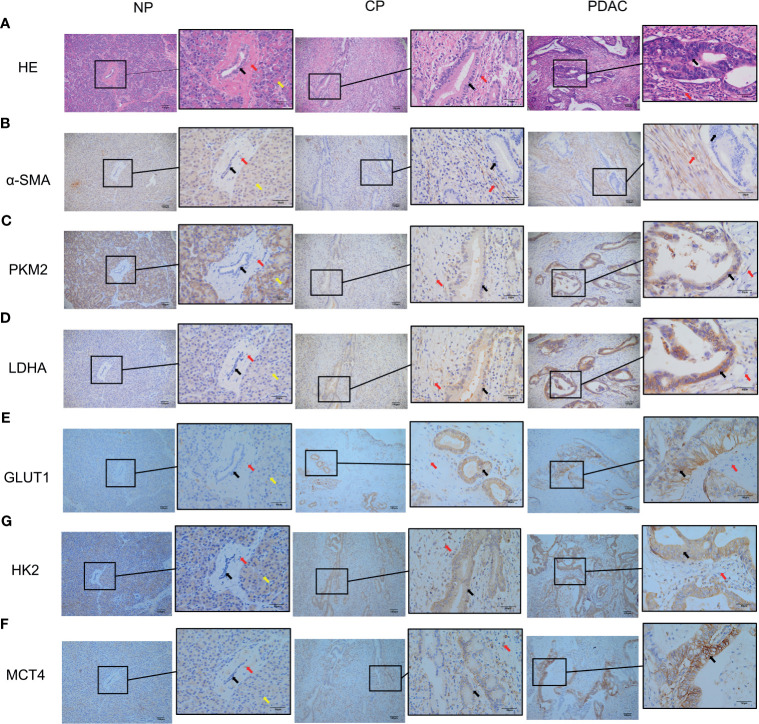
Immunohistochemistry showed that the α-SMA and glycolysis markers in the pancreatic tissues of humans in the normal group, CP group and PDAC group gradually increased (100X and 400X; black arrow: cancer/normal epithelial duct cells; red arrow: stromal cells (fibroblasts, PSCs); yellow arrow: acinar cells). **(A)** HE in normal/CP/PDAC human pancreas; **(B)** α-SMA in normal/CP/PDAC human pancreas; **(C)** PKM2 in normal/CP/PDAC human pancreas; **(D)** LDHA in normal/CP/PDAC human pancreas; **(E)** GLUT1 in normal/CP/PDAC human pancreas; **(F)** HK2 in normal/CP/PDAC human pancreas; **(G)** MCT4 in normal/CP/PDAC human pancreas.

In immunohistochemical sections of human tissues, we observed findings similar to those in the animal model, namely, activation of PSCs and enhancement of glycolytic metabolism in CP and PDAC tissues (**Figures 6A–G**). We analysed the correlation between α-SMA in the peripancreatic duct interstitium and glycolysis marker expression in pancreatic duct epithelial cells in the CP and PDAC groups. We first confirmed that the expression of α-SMA in the CP group and PDAC group was significantly different from that in the normal pancreas group (P<0.01, P<0.001) (**Table 4**). Correlation analysis (**Table 5**) showed that there was a correlation between PKM2 (r=0.459, P<0.05)/LDHA (r=0.457, P<0.05)/GLUT1 (r=0.567, P<0.05)/HK2 (r=0.535, P<0.05)/MCT4 (r=0.630, P<0.01) expression in pancreatic duct epithelial cells and α-SMA expression in the peri-pancreatic duct interstitium in the CP groups; there was also a correlation between PKM2 (r=0.622, P<0.01)/LDHA (r=0.688, P<0.01)/GLUT1 (r=0.515, P<0.01)/HK2 (r=0.614, P<0.01)/MCT4 (r=0.412, P<0.01) expression in pancreatic duct epithelial cells and α-SMA expression in the peri-pancreatic duct interstitium in the PDAC groups.

**Table 4:** Differential expression of α-SMA in CP/PDAC tissues and normal Pancreatic tissues.

	α-SMA expression		Total	P-value
	Low	High		
CP	5(26.3%)	14(73.7%)	19	<0.01^†^
PDAC	5(12.5%)	35(87.5%)	40	<0.001^†^
Normal pancreas	16(80.0%)	4(20.0%)	20	

Differential expression of α-SMA in CP/PDAC tissues and normal Pancreatic tissues was compared using the χ^2^ test. †: Compared with normal Pancreatic tissues.

**Table 5:** Correlations of α-SMA expression with glycolysis markers (PKM2, LDHA, GLUT1, HK2 and MCT4) in CP/PDAC tissues.

	Immunoreactivity	α-SMA expression		R-value	P-value
		Low	High		
CP	PKM2 expression				
	Low	4	4	0.459	<0.05
	High	1	10		
PDAC	PKM2 expression				
	Low	4	3	0.622	<0.01
	High	1	32		
CP	LDHA expression				
	Low	3	2	0.457	<0.05
	High	2	12		
PDAC	LDHA expression				
	Low	4	2	0.688	<0.01
	High	1	33		
CP	GLUT1 expression				
	Low	5	5	0.567	<0.05
	High	0	9		
PDAC	GLUT1 expression				
	Low	5	9	0.515	<0.01
	High	0	26		
CP	HK2 expression				
	Low	4	3	0.535	<0.05
	High	1	11		
PDAC	HK2 expression				
	Low	5	6	0.614	<0.01
	High	0	29		
CP	MCT4 expression				
	Low	5	4	0.630	<0.01
	High	0	10		
PDAC	MCT4 expression				
	Low	4	8	0.412	<0.01
	High	1	27		

Correlations of α-SMA expression with glycolysis markers (PKM2, LDHA, GLUT1, HK2 and MCT4) in CP/PDAC tissues were evaluated by Spearman’s rank correlation.

## Discussion

Quiescent PSCs in normal tissues play an important role in the metabolic homeostasis of pancreatic ECM by regulating ECM-related enzymes. PSCs are activated during pancreatic tissue injury and stress and acquire a myofibroblast phenotype. Lipid droplets rich in vitamin A disappear in their cytoplasm. With the expression of α-smooth muscle actin (α-SMA) as a marker, a large amount of extracellular matrix (ECM) is synthesized, and a variety of cytokines are secreted (10). Activated PSCs have contractile microfilament-like structures, which can help them migrate to the injured area and secrete ECM to promote pancreatic tissue repair. When the stimulating factor persists, the synthesis of ECM exceeds its degradation, so pancreatic fibrosis occurs (11). Pancreatic stellate cells are currently considered to be the initiators of chronic pancreatitis and pancreatic cancer. Studies have shown that activated PSCs in the extracellular matrix of pancreatic cancer are cancer-associated fibroblasts (CAFs). CAFs are activated fibroblasts and an important part of tumor stromal cells (19–21). Through specific communication with cancer cells, CAFs directly promote tumor initiation, progression, and metastasis (22, 23).

In this study, we successfully obtained activated and quiescent PSCs from pancreatic cancer patients and individuals with a normal pancreas using a method described previously, and phenotypic identification was performed by immunofluorescence. In our previous work, we found that activated PSCs promoted the proliferation, migration and invasion of pancreatic cancer cells (24). We replicated the results using activated cells isolated in this study. Subsequently, we co-cultured activated PSCs with HPNE cells, a normal pancreatic duct epithelial cell line that is thought to have the potential to differentiate into cancer cells (25), and found that activated PSCs also promoted the proliferation, invasion, and migration of HPNE cells. When CP occurs, there are activated PCSs around benign pancreatic duct epithelial cells, and chronic pancreatitis is a precancerous lesion. Does this phenomenon suggest that activated PSCs have a role in promoting the malignant transformation of CP? Studies have shown that chronic pancreatitis is the result of the combined action of cytokines, inflammatory cells and activation of pancreatic stellate cells, among which PSCs are considered to be the target of various cytokines in chronic pancreatitis and the core of the disease progression of CP (26). In summary, PSCs are closely related to the occurrence and development of chronic pancreatitis and pancreatic cancer, but their role in the carcinogenesis of chronic pancreatitis has not yet been elucidated.

As a characteristic of cancer cells, the Warburg effect is a very obvious feature of benign cells after malignant transformation. Compared with normal cells, tumor cells exhibit a higher glucose metabolic rate and preferentially utilize glycolysis over oxidative phosphorylation, even when oxygen levels are adequate (15). At present, some studies have confirmed that CAFs are involved in regulating and promoting the glucose metabolism of cancer cells. Shan et al. (16) found that CAFs could promote the glucose metabolism of pancreatic cancer cells and enhance their migration and invasion ability. Zhao et al. (17) found that exosomes secreted by CAFs can regulate glucose metabolic reprogramming of pancreatic cancer cells through the KRAS pathway and enhance their invasion and metastasis abilities. Therefore, will PSCs enhance glycolytic metabolism in CP and eventually lead to malignant transformation of CP? We tentatively detected the changes in glycolysis markers and glucose metabolic capacity of HPNE after co-culture with PSCs. The results showed that co-culture with CaPSCs significantly promoted the expression of the glycolysis marker PKM2/LDHA/GLUT1/HK2/MCT4 (including mRNA level) in HPNE cells, and concurrently, lactic acid production and glucose consumption were also significantly increased after co-culture. These results suggest that activated PSCs may enhance the Warburg effect in HPNE cells to promote their malignant transformation. Subsequently, we tested this idea *in vivo*. We established rat models of CP and PDAC using drug induction. To verify the relationship between α-SMA in PSCs and glycolysis markers in pancreatic duct epithelial cells in the same region, paraffin sections of CP and PDAC tissues were obtained for immunohistochemical identification of α-SMA and glycolysis markers (PKM2, LDHA, GLUT1, HK2 and MCT4), respectively, and compared with those of normal pancreas. The results showed that the expression of α-SMA in both pancreatic duct cells and surrounding stromal cells was low in normal pancreas, but the expression of α-SMA in pancreatic duct epithelial cells and surrounding stromal cells was increased in chronic pancreatitis, and this increase was more obvious in pancreatic cancer. The results showed that the expression of glycolysis markers in pancreatic duct cells and α-SMA in surrounding stromal cells was low in normal pancreatic tissues, but the expression of these proteins was increased in CP tissues, and the increase was more pronounced in PDAC tissues. Then, we collected pathological sections from clinical patients and explored the correlation between α-SMA and PKM2/LDHA/GLUT1/HK2/MCT4 in heterogeneous pathological sections of human CP and PDAC. Unlike animal models that allow for the strict control of variables, clinical patients themselves have wide-ranging differences among each individual, while patients with chronic pancreatitis and pancreatic cancer have greater pathological tissue heterogeneity. This is because chronic pancreatitis and pancreatic cancer usually have abundant matrix components, and PSCs, as the main component in the matrix, have rich heterogeneity, which further enhances the heterogeneity of chronic pancreatitis and pancreatic cancer. This characteristic of heterogeneity makes the results of correlation analysis more practical (27, 28). Compared with that in normal pancreatic tissue, α-SMA expression in the matrix of human CP and PDAC tissue was increased significantly. The correlation between the glycolysis markers of pancreatic duct cells and the activation of pancreatic stellate cells in the surrounding matrix was observed in both chronic pancreatitis and pancreatic cancer. This suggests an association between activated PSCs and the Warburg effect in pancreatic duct epithelial cells.

The overactivation of PSCs maintains and promotes pancreatic fibrosis, leading to the occurrence and development of chronic pancreatitis. The above experimental results indicate that PSCs have the ability to enhance the glycolysis level of CP. How does the activated PSC regulate the glycolytic effect of CP and ultimately lead to the malignant transformation of CP? In addition to the aforementioned enhancement of the Warburg effect of CAF-derived exosomes on pancreatic cancer cells, some cytokines secreted by CAFs also have this effect. It has been shown that hepatocyte growth factor secreted by tumor stromal CAFs can increase glycolysis levels in head and neck squamous cell carcinoma and pancreatic cancer cells by activating c-Met (29, 30). In breast cancer, CAFs secrete a variety of cytokines, including CXCL10, IL6, and IL8, which promote glycogenolysis and inhibit glycogen synthesis in breast cancer cells, ultimately leading to an increase in glycolysis (31). CAFs can enhance the Warburg effect of tumors through a variety of paracrine signals, which guides the study of the mechanism by which PSCs enhance the Warburg effect in CP. Another key mediator of the Warburg effect is hypoxia-inducible factor-1α (HIF-1α) (32). Hypoxia is common in fast-growing solid tumors, and Hif-1α is a key factor that helps tumor tissues adapt to the hypoxic microenvironment (33, 34). Recent studies have shown that HIF-1α can be activated by a variety of other factors even under normoxic conditions and mimic many hypoxia-mediated phenomena, resulting in a state of “pseudohypoxia” (35). The glycolysis process in tumors can be driven by active HIF-1α, regardless of whether there is hypoxia. HIF1α can increase the activity of a series of enzymes related to glycolysis and metabolism (32, 36). Several studies have confirmed the role of HIF-1α in promoting glycolysis in pancreatic cancer (37–39). GLUT1 and MCT4, two glycolytic metabolic enzymes closely associated with hypoxia and HIF-1α, were significantly increased in CP in the clinical samples investigated in this study. Exploring whether hypoxia and Hif-1α activation exist stably in CP and the relationship between them and PSCs will help uncover the mechanism of transformation from chronic pancreatitis to pancreatic cancer. KRAS mutation also deserves attention in the study of the carcinogenesis of CP. KRAS mutations occur at all stages from pancreatic intraepithelial neoplasia (PanINs) to invasive and metastatic disease and can be observed in more than 90% of pancreatic cancer patients (40). It is worth noting that the KRAS mutation is also associated with the upregulation of HIF-1α and the Warburg effect in cancer cells (41–43). Several studies on tumor-stromal interactions in pancreatic cancer have suggested that PSCs and KRAS mutations have mutual regulation (44–46). In view of the presence of KRAS mutations in most pancreatic cancers, whether PSCs enhance the Warburg effect through mutual regulation with KRAS mutations and ultimately lead to the carcinogenesis of CP is worthy of further study.

**Figure 7:**
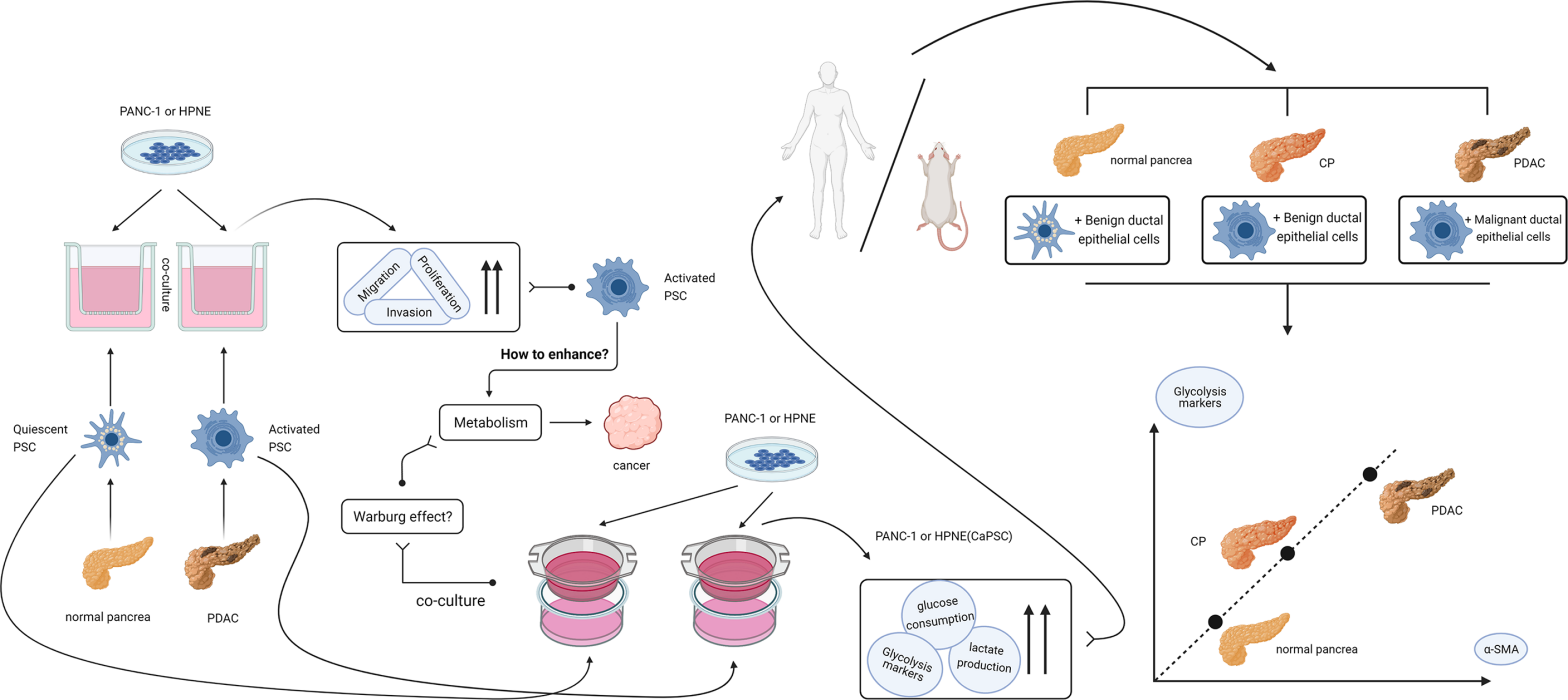
Design idea and verification flow chart of this study. After finding that activated PSCs of pancreatic cancer origin can promote the proliferation, migration and invasion of benign and malignant pancreatic duct epithelial cells, we hypothesized that activated PSCs may promote the malignant transformation of chronic pancreatitis. In the process of searching for the mechanism, a series of experiments confirmed that the activated PSCs could promote the Warburg effect in benign pancreatic duct epithelial cells *in vitro*, and the correlation between the glycolytic proteins of pancreatic duct cells and the activated PSCs in the peri-pancreatic duct matrix was confirmed *in vivo*.

In conclusion, we believe that activated pancreatic stellate cells have the ability to enhance the Warburg effect in pancreatic duct epithelial cells and play a supporting and promoting role in the malignant progression of chronic pancreatitis. The idea and process of the whole experiment are shown in **Figure 7**. The specific influencing mechanism needs to be further studied. Our current study provides a new theoretical basis for further understanding the mechanism of CP malignancy and selecting diagnosis and treatment targets for reversing CP malignancy.

## Data Availability Statement

The original contributions presented in the study are included in the article/supplementary material. Further inquiries can be directed to the corresponding authors.

## Ethics Statement

The studies involving human participants were reviewed and approved by Ethics Committee of the Anhui Provincial Hospital of the First Affiliated Hospital of the University of Science and Technology of China. The patients/participants provided their written informed consent to participate in this study. The animal study was reviewed and approved by Ethics Committee of the Anhui Provincial Hospital of the First Affiliated Hospital of the University of Science and Technology of China.

## Author Contributions

QH and FM contributed to the conception and design, as well as critical revision of the article for important intellectual content. YT and FS conceived and designed the experiments, performed the experiments and analyzed and interpreted the data, drafted and revised the manuscript. MC, ZL, and YP analyzed and interpreted the data. All authors contributed to the article and approved the submitted version.

## Funding

This work was supported by funds from Anhui Provincial Natural Science Foundation (2008085QH416 and 2008085QH419) and New Medical Association of University of science and technology of China (WK9110000105).

## Conflict of Interest

The authors declare that the research was conducted in the absence of any commercial or financial relationships that could be construed as a potential conflict of interest.

## Publisher’s Note

All claims expressed in this article are solely those of the authors and do not necessarily represent those of their affiliated organizations, or those of the publisher, the editors and the reviewers. Any product that may be evaluated in this article, or claim that may be made by its manufacturer, is not guaranteed or endorsed by the publisher.
